# Effects of Dietary Rapeseed Meal on Growth Performance, Carcass Traits, Serum Parameters, and Intestinal Development of Geese

**DOI:** 10.3390/ani11061488

**Published:** 2021-05-21

**Authors:** Zhenming Fu, Guoqiang Su, Haiming Yang, Qingyu Sun, Tao Zhong, Zhiyue Wang

**Affiliations:** 1 College of Animal Science and Technology, Yangzhou University, Yangzhou 225009, China; dyjiajiehuagong@163.com (Z.F.); sgqyzu@163.com (G.S.); sqy203610@163.com (Q.S.); zhong19825305405@163.com (T.Z.); dkwzy@263.net (Z.W.); 2 Joint International Research Laboratory of Agriculture and Agri-Product Safety of Ministry of Education of China, Yangzhou University, Yangzhou 225009, China

**Keywords:** rapeseed meal, geese, performance, carcass traits, serum parameters, intestinal development

## Abstract

**Simple Summary:**

The poultry industry faces challenges such as a shortage of protein feed and increased feed cost. Compared to soybean meal, rapeseed meal (RSM) is a cheaper protein source that can be used in poultry diets. China is both the largest RSM and geese producer in the world. Therefore, the active use of RSM for geese diets can contribute to the development of poultry production. We found that geese had a good adaptation to the diet containing 16% dietary RSM through the investigation.

**Abstract:**

The use of inexpensive nonconventional feed materials, such as rapeseed meal (RSM), could help alleviate the shortage of feed materials in the poultry industry. This study was to investigate the effects of dietary double-low RSM on growth performance, carcass traits, serum parameters, and intestinal development of geese. A total of 270 healthy 35-day-old male Jiangnan White geese were randomly divided into five treatments, with six replicate pens of nine geese each. The geese were fed five isonitrogenous and isocaloric diets containing 0%, 4%, 8%, 12%, and 16% RSM replacing dietary soybean meal for 35 days. At 35, 49, and 70 d, the BW and feed intake were recorded. All Samples were collected at 70 d of age. The results showed that dietary RSM up to 16% did not affect the BW, ADFI, ADG, and feed/gain ratio (F/G) during 35 to 49 d, 49 to 70 d, and 35 to 70 d periods (*p* > 0.05). At 70 d, no difference was observed in carcass yield or serum biochemical parameters among groups (*p* > 0.05). Dietary 12% and 16% RSM significantly increased the concentration of serum GH compared with 0%, 4%, 8% groups (*p* < 0.01), but serum TSH, T3 and T4 were unaffected (*p* > 0.05). The relative weights of heart, liver, spleen, proventriculus, gizzard, and small intestine were similar among groups (*p* > 0.05). However, the geese fed dietary 16% RSM had greater bursa of Fabricius than geese in the 8% group (*p* < 0.05). Intestinal morphology was unaffected by treatments (*p* > 0.05). According to the findings, dietary RSM up to 16% can be used in geese diets without impact on production performance.

## 1. Introduction

With the acceleration of the intensive geese breeding process in China, the geese industry directly competes with humans for major feed ingredients. The shortage of protein source feeds (e.g., soybean meal, SBM) required to formulate balanced geese ration increased annually, together with unit cost. Feed cost is a great expense accounting for about 70% of the total cost of geese production, of which the protein source represents a large part [1,2]. Thus, the discovery and utilization of more cost-effective alternatives that maintain normal growth performance and are preferably not used in human nutrition would promote geese production.

Rapeseed meal (RSM), an oil-byproduct of high yield, is a cheaper alternative source of plant protein (crude protein, 35–42%) for poultry diets compared with SBM [3]. It is also rich in vitamins (e.g., thiamine, choline, and folic acid), minerals (e.g., calcium, selenium, zinc, and iron), and avian limiting amino acid (e.g., methionine and lysine) [4,5]. However, the use of RSM in monogastric animal nutrition is mainly limited by the presence of glucosinolates (GLS) and its toxic metabolites of enzymatic hydrolysis [6]. The high concentration of GLS from excessive inclusion of dietary RSM could lead to hypothyroidism, abnormalities in digestive function, liver and kidney disorders, and impairment to body health [7,8,9]. In addition, anti-nutritional factors (ANFs) of RSM—including phytate, sinapine, condensed tannin (CT), and non-starch polysaccharides (NSP)—would decrease feed palatability, metabolism of feed nutrients [10], and thus growth performance [11].

Variety breeding and improvement, and seed processing by prepress solvent extraction or expeller-extraction method, contribute to reducing ANFs in RSM [12,13,14] and promoting RSM application in poultry feed. Qin et al. [9] found that meat ducks fed 6.6% of dietary RSM (7.75 μmol GLS/g) exhibited similar organ health and growth rate compared with diets based on corn–soybean meal from 15 to 35 d of age. Ahamed et al. [15] reported that canola meal can be used as up to 20% of the starter (1 to 28 d) and finisher (29 to 42 d) diets without having any adverse effects of broiler performance. Turkeys receiving 6% to 18% of dietary RSM maintained normal body weight after a 147-d feeding trial [16]. These suggested that moderate inclusion of low-GLS RSM could effectively substitute SBM in diets for the mentioned poultry. In contrast, the literary data on feeding values of RSM is scarce on geese. The deleterious effects of GLS partly depend on strain and age of birds and duration of feeding [3,16]. The physiological structure, digestive function, and growth period of geese are not the same as those of other poultry (e.g., broilers and turkeys) [13,16,17] lead to a difference in tolerance of GLS and utilization efficiency of RSM-contained diets. Taken together, the basic hypothesis was that geese could utilize RSM, but with an uncertain amount. Therefore, this study aimed to investigate the effects of dietary levels of RSM as a replacement for SBM on growth performance, carcass traits, serum parameters, and intestinal development of geese.

## 2. Materials and Methods

### 2.1. Experimental Design, Diets, and Management

All procedures of this study were permitted by the Institutional Animal Care and Use Committee (IACUC) of the Yangzhou University Animal Experiments Ethics Committee, with the permit number: SYXK (Su) IACUC 2012-0029.

This experiment was conducted at the experimental ranch of Yangzhou University (Yangzhou, China) from June to August in 2020. A total of 270 healthy 35-day-old male Jiangnan White geese from the same hatch were obtained from a commercial hatchery (Jiangsu Lihua Animal Husbandry Co., Ltd., Changzhou, Jiangsu Province, China). According to a completely randomized design, the geese were all of similar body weight (BW) and randomly assigned to five dietary treatments, with six replicate pens per treatment and nine geese per pen. The control group was fed a basal corn-soybean meal (corn-SBM) diet, and the four experimental groups were fed diets containing 4%, 8%, 12%, or 16% rapeseed meal (RSM) to replace the main protein source (SBM) in the control diet. These diets (mash form) were formulated to be isonitrogenous and isocaloric and meet or exceed the nutrient requirements of geese according to the NRC (1994) and prior research results from our laboratory [1,2,18]. Before diet formulation, all energy sources and protein-containing ingredients (i.e., corn, RSM, SBM, rice husk, and wheat bran) were analyzed in duplicate for dry matter (DM), crude protein (CP), crude fat (EE), crude fiber (CF), calcium (Ca), total phosphorus (TP), and ash according to the classical procedures set forth by the Association of Official Analytical Chemists (AOAC, 2006) [19]. Methionine (Met) and lysine (Lys) were determined using a Hitachi L-8900 automatic amino acid analyzer (Hitachi Ltd., Tokyo, Japan). The composition and nutrient levels of the experimental diets are listed in Table 1. The RSM sample was also analyzed for GLS content by an Agilent 1290 Infinity-II high-performance liquid chromatography (HPLC) system (Agilent Technologies Inc., Santa Clara, CA, USA). The chemical composition of RSM is shown in Table 2. The mentioned feed ingredients were crushed and mixed using a hammer mill (2.5-mm screen, model SWFP66×60C, Muyang, Yangzhou, China) before producing mash diets. During the 35-days experiment trial, all geese were reared in plastic wire-floor pens (2.28 × 1.24 m) and exposed to natural light. The room temperature was approximately 28 °C at the start and gradually decreased to approximately 23 ± 2 °C (day and night) by temperature control equipment including fans and wet curtains during. Water and feed were provided ad libitum.

### 2.2. Growth Performance

The growth performance of geese was evaluated in terms of body weight (BW), average daily feed intake (ADFI), average daily gain (ADG), and the feed/gain ratio (F/G). The BW and feed intake of the birds in each pen were recorded at 35, 49, and 70 days of age. The ADG, ADFI, and F/G from 35 to 49, 49 to 70, and 35 to 70 d were calculated.

### 2.3. Sample Collection and Measurement

At 70 d of age, all geese were weighed individually after fasting for 6 h. One goose of each pen with the mean weight of the pen was selected. Duplicate blood samples (3 mL × 2) were collected in sterile procoagulant tubes via wing venipuncture, centrifuged at 2000× *g* for 10 min at 4 °C to harvest serum, and stored at −20 °C until analysis of serum parameters. Following this, the geese were exsanguinated by severing the jugular vein and carotid artery on one side of the neck. After bleeding and plucking (soaking in 70 °C water for approximately 2 min), the weight was recorded. Then, the geese were eviscerated, and the semi-eviscerated carcass, eviscerated carcass, heart, liver, spleen, pancreas, bursa of Fabricius, proventriculus, empty gizzard, breast muscle, thigh muscle, and abdominal fat were weighed. The duodenum (from the gizzard outlet to the end of the pancreatic loop), jejunum (from the pancreatic loop to Meckel’s diverticulum), and ileum (from Meckel’s diverticulum to the ileo-caeco-colic junction) were isolated and weighed after evacuation. The percentages of the small intestine, carcass, semi-eviscerated carcass, and eviscerated carcass were calculated relative to the live BW, while the percentages of organs, breast muscle, thigh muscle, and abdominal fat were calculated relative to the eviscerated carcass weight.

### 2.4. Serum Biochemical and Hormonal Parameters

Serum concentrations of total protein (TP), albumin (ALB), glucose (GLU), triglyceride (TG), cholesterol (CHO), blood urea nitrogen (BUN), high-density lipoprotein (HDL), low-density lipoprotein (LDL), calcium (Ca), phosphorus (P), glutamic pyruvic transaminase (ALT), glutamic-oxalacetic transaminase (AST), and alkaline phosphatase (AKP) were measured using a UniCel DxC 800 Synchron Clinical System autoanalyzer (Beckman Coulter Inc., Fullerton, Canada) with the respective detection kits (Shengkang Bio-tech Co., Ltd., Shaoxing, China). Serum TP was assayed by the biuret method. Serum ALB was assayed by the microplate assay method. Serum GLU was assayed by the GLU oxidase method. Serum BUN was assayed by the rate method. Serum TG and CHO were assayed by the enzyme-colorimetric method. Serum HDL and LDL were assayed by the direct method. Serum Ca and P were assayed by the ion electrode method. Serum ALT and AST were assayed by continuous monitoring method. Serum AKP was assayed by the enzymatic method. Serum growth hormone (GH), thyroid-stimulating hormone (TSH), triiodothyronine (T3), and tetraiodothyronine (T4) were determined by the ELISA method with the corresponding ELISA kits (Ybio Bio-tech Co., Ltd., Shanghai, China).

### 2.5. Intestinal Morphology

At 70 d of age, another goose of each pen with the mean weight of the pen was selected. After slaughtering by exsanguination, the duodenum, jejunum, and ileum were isolated. Sections (approximately 2 cm in length) from the midpoints of the duodenum, jejunum, and ileum were excised and flushed with phosphate-buffered saline (PBS) and immediately fixed by placing them in 10% neutral-buffered formalin solution. Each fixed sample was embedded in wax, sectioned at a thickness of 5 μm, and stained with hematoxylin-eosin. For histological morphometric observations, the slides were analyzed by light microscopy, and digital images were captured (LY-WN-HP SUPER CCD, Chengdu, China). The height of villi and depth of crypts were measured for 10 villi at ×40 magnification as described by Lu et al. (2011) [17].

### 2.6. Statistical Analysis

All the data were initially processed using Excel 2019 and analyzed using SPSS 20.0 (SPSS Inc., Chicago, IL, USA, 2011). One-way ANOVA with a post hoc test was used to elucidate significant differences. All the data were checked for normality. Duncan’s test was used for multiple comparisons when a significant difference was detected. Differences were considered to be statistically significant at *p* < 0.05, and 0.05 < *p* < 0.10 was considered to be a trend towards significance. All the data were presented as the mean and standard error of the means (SEM).

## 3. Results

### 3.1. Growth Performance

BW, ADFI, ADG, and the F/G of geese fed graded levels of dietary RSM are shown in Table 3. Diets containing 4%, 8%, 12%, and 16% RSM did not affect the BW (40 d and 70 d of age) of geese compared with the control group (*p* > 0.05). During 35 to 49 d, 49 to 70 d, and 35 to 70 d of age, similar ADFI, ADG, and F/G of geese were observed among groups (*p* > 0.05).

### 3.2. Carcass Traits

As shown in Table 4, different levels of dietary RSM did not affect slaughter performance of geese at 70 d of age, including slaughter yield, half-eviscerated carcass yield, eviscerated carcass yield, breast yield, thigh yield, or abdominal fat yield (*p* > 0.05).

The effect of dietary RSM on visceral development of the geese at 70 d is shown in Table 5. No significant difference was observed in the relative weights of heart, liver, spleen, proventriculus, or gizzard among groups (*p* > 0.05). However, the geese fed dietary 16% RSM had greater bursa of Fabricius than those of geese in the 8% group (*p* < 0.05).

### 3.3. Serum Biochemical and Hormonal Parameters

The effect of dietary RSM on serum biochemical parameters of the geese at 70 d are shown in Table 6. Different levels of dietary RSM had no significant effect on serum concentrations of TP, ALB, GLU, TG, CHO, BUN, HDL, LDL, Ca, P, ALT, AST, or AKP (*p* > 0.05).

The serum hormonal parameters of geese at 70 d are shown in Table 7 (*p* > 0.05). The serum concentration of GH significantly increased when geese were fed 12% and 16% dietary RSM (*p* < 0.05). The serum concentration of T3 of geese fed 8% dietary RSM slightly increased compared with groups 4% (*p* = 0.051). Serum TSH and T4 were unaffected by dietary RSM (*p* > 0.05).

### 3.4. Intestinal Development

The relative weight and morphological measurements of the small intestine in geese are presented in Table 8. Dietary RSM up to 16% had no effect on the relative weight of duodenum, jejunum, and ileum (*p* > 0.05). Dietary RSM of 12% and more slightly decreased the crypt depth of duodenum (*p* = 0.06), whereas no difference was found in villus height among groups (*p* > 0.05). No effects of dietary RSM were observed on the crypt depth or the villus height of the jejunum or ileum (*p* > 0.05).

## 4. Discussion

### 4.1. Chemical Composition of Rapeseed Meal

The glucosinolate concentration in the rapeseed meal used in the current study, confirmed the standard of low-glucosinolate RSM (GLS ≤ 45 μmol/g) promulgated by the Ministry of Agriculture of China. The content of CP, CF, ash, Ca, TP, Met, and Lys agreed well with the data in Tables of Feed Composition and Nutritive Values in China [20]. Compared with SBM, RSM contained less CP (36.73% vs. 43.01%), and Lys (1.63% vs. 2.66%), but more CF (9.48% vs. 6.48%) and Met (0.71% vs. 0.58%). Therefore, the RSM selected was representative and usability.

### 4.2. Growth Performance

During the entire experiment trial, no geese exhibited clinical symptoms such as diarrhea, leg abnormalities, or sudden death due to low-glucosinolate RSM treatment. Although these adverse reactions caused by excessive GLS are likely to occur in chickens [6,21], they have not been pronouncedly reported in geese yet. Further study was significantly needed on the toxicity of GLS on geese. However, it was reported that chickens could positively respond to dietary double-low RSM (e.g., Canola, up to 20%) and grow without abnormal symptoms [11]. In view of the fact that the current RSM variety contains low GLS, the pathogenic impact tends to be minor [14].

Previous research reported that dietary RSM from 15% to 20% had no negative effect on broiler performance [8,15,22,23]. The current study also indicates that dietary RSM could maintain the growth performance of geese over short-term feeding. However, some other studies [11,24] found that adding 15% or 20% RSM to diets reduced weight gain and increased F/G of broilers. In view of different feeding designs, the discrepancy in growth depression may partly depend on feed factors including RSM varieties, processing, and diets formulation, determining the dietary GLS and ANFs contents [25,26].

Additionally, Zhu et al. [3] demonstrated that the BW gain of Cherry Valley ducks decreased linearly as the dietary RSM increased from 5% to 20% (7 to 14 d of age) growth rate was not affected from 15 to 21 d. Mikulski et al. [16] observed that turkeys receiving dietary RSM up to 18% had an increased F/G during a long feeding period of 147 d. In the current study, the development of the digestive tract of 35 to 70-d-old geese was improved after a brooding period of 28 d. As raised for a comparatively shorter period than turkeys, the negative reaction of geese growth to dietary RSM and its ANFs might be smaller than that in the starter period. The current results likely indicate a low hypotoxicity of the selected RSM variety and distinct adaptability of geese to graded levels of dietary RSM. In view of the positive feeding response, rapeseed meal is a suitable protein substitute for SBM in geese diets.

### 4.3. Carcass Traits

Carcass characteristic is one of the most crucial indicators of growth performance and saleability of meat poultry [2]. The reported effects of RSM on carcass yield of poultry were not consistent in recent studies. Mikulski et al. [16] observed that dietary RSM from 6% to 18% had no effect on dressing percentage, breast and thigh yield, and the abdominal fat pad of turkeys, as is similar to the current study. Moreover, in broilers feeding, it was generally suggested that diets containing 20% of low-glucosinolate RSM did not affect carcass and breast yields negatively [15,23]. In addition, the abdominal fat pad decreased linearly when dietary RSM increased from 4% to 16% [27]. However, Taraz et al. [28] observed that the replacement of high-glucosinolate RSM (79 μmol/g RSM) with SBM at a level greater than 7.5% in diet reduced carcass weight and the abdominal fat pad of broiler chicks, as the feed intake decreased followed by a poor growth rate. For other crop byproducts, Yu et al. [2] reported that replacing low-gossypol cottonseed meal for dietary SBM could maintain the carcass yield of geese. Following Yu et al., the current result may suggest that geese fed low-glucosinolate RSM-contained diets can achieve slaughter performance as well as those fed corn-SBM-contained diets.

The relative weight of visceral organs is a key indicator of organ health and physical functions. The toxicity of RSM may stunt the organogenetic development of animals [7]. Supported by serum AKP, ALT, and AST, however, the current result was agreed with Qin et al. [9], who found no visible changes on the liver of ducklings fed RSM up to 30% for 21d. Although Zhu et al. [3] observed liver enlargement of ducks receiving 5% to 20% dietary RSM with 1.37 to 5.31 μmol GLS/g diet, it was argued that a short period of feeding double-low RSM was insufficient to induce severe liver damage. In contrast, long-term feeding of dietary RSM causes organic changes by ingested GLS and promotes gizzard enlargement by its high content of crude fiber and NSP [6,15,16]. In the current study, however, all diets provided equal amounts of dietary fibers. Moreover, geese have developed gizzards which lead to high endurance to dietary fibers [29]. This can explain why the current gizzards similarly weighed at the end of the feeding period. As inconsistent with Zhu’s et al. [3] study, however, the enlarged bursa of Fabricius (BF) manifests that the geese could exhibit an immune reaction to high dietary RSM even under low ingestion of GLS (<1.97 μmol GLS/g diet). Nutritional research suggested that the enlargement of immune organs commonly represents a positive immune response to feed ingredients or dietary nutrients, which indicated that immune function was enhanced [30]. However, the visible enlargement accompanied by pathological damage could also be found in immune organs (e.g., spleen) when poultry was impacted by pathogenic factors [31]. Although no distinctive lesion was observed on the BF, the current data are not sufficient to prove the correlation between the BF ratio and immune status. To sum up, short-term use of low-glucosinolate RSM presents no impairment to circulatory and alimentary organ health and functions of geese.

### 4.4. Serum Biochemical and Hormonal Parameters

Serum parameters reflect the metabolism of nutrients and physiological status in the body regarding growth and development. Serum biochemical parameters indicate that dietary RSM did not affect the metabolism of proteins, lipids, and minerals, which supported the current growth performance. It was well-acknowledged that GH is an important factor in body growth and metabolism. However, research on chickens [32] showed that serum GH was not simply positively correlated with growth performance. A 35-d RSM feeding did not increase or decrease the growth rate of geese. As there was no research to prove the correlation between dietary RSM and GH, further study would be needed.

Dietary GLS has been shown to suppress the synthesis of thyroid hormone then cause the pituitary to release TSH, which induce thyroid follicular hyperplasia and goiter. However, an elevation of serum triiodothyronine even occurred through feeding RSM. The same result was also achieved by Qiao and Classen [33] that solvent-extracted canola meal increased serum triiodothyronine levels in the broiler. Moreover, Woyengo et al. [13] and Zhu et al. [3] found no difference in serum thyroid hormone of broilers or ducks, respectively. Although the mechanism of increasing serum triiodothyronine by dietary RSM is still unclear, the current result can at least show that geese fed low-GLS RSM for 35 d can maintain the normal endocrine function of the thyroid gland.

### 4.5. Intestinal Development

Intestinal morphological structure partly determines the nutrient digestion and absorption in geese. Dietary RSM could influence growth performance by altering intestinal morphology [34]. As reported by Gopinger et al. [8], the results obtained showed a tendency of a linear reduction of crypt depth of duodenum with the inclusion of canola meal in the diet, which is similar to the current findings. Higher villus height and shallower crypt depth commonly indicate highly mature cells and greater secretory ability [35]. Though the morphological observation aligns with geese performance, the potential effect of RSM on intestinal development needs further evaluation.

## 5. Conclusions

During a 35-d feeding, a dietary rapeseed meal from 4% to 16% had no obvious effect on the growth performance, carcass traits, and intestinal development of geese from 35 to 70 d of age. We recommend that rapeseed meal can be used up to 16% in the geese diet from 35 to 70 d of age for saving feed cost.

## Figures and Tables

**Table 1:** Composition of rapeseed meal and soybean meal (air-dry basis) ^1^.

Items (%)	Rapeseed Meal	Soybean Meal
Moisture	9.74	11.36
Crude protein	36.72	43.01
Crude fat	9.48	1.93
Crude fiber	7.08	6.48
Calcium	0.74	0.32
Total phosphorus	1.16	0.58
Methionine	0.71	0.58
Lysine	1.63	2.65
Glucosinolate (μmol/g)	12.29	–

^1^ Measured value. – Not measured.

**Table 2:** Composition and nutrient levels of experimental diets (air-dry basis) %.

Items	Groups ^1^				
	Control	RSM_4_	RSM_8_	RSM_12_	RSM_16_
Corn	58.33	59.02	59.71	60.40	61.09
Soybean meal (CP 43.01%)	25.40	22.36	19.31	16.27	13.22
Rapeseed meal (CP 36.72%)	0.00	4.00	8.00	12.00	16.00
Rice husk	7.51	7.22	6.94	6.66	6.38
Wheat bran	5.26	3.94	2.63	1.31	0.00
Limestone	1.08	1.07	1.07	1.07	1.07
Dicalcium phosphate	1.01	0.95	0.89	0.82	0.76
Salt	0.30	0.30	0.30	0.30	0.30
DL-methionine	0.13	0.12	0.11	0.10	0.09
L-lysine HCL	0.00	0.02	0.05	0.07	0.09
Premix ^2^	1.00	1.00	1.00	1.00	1.00
Total	100.00	100.00	100.00	100.00	100.00
Nutrient level ^3^, %					
ME (MJ/kg)	10.98	10.98	10.98	10.98	10.98
Crude protein	16.54	16.54	16.54	16.54	16.54
Crude fiber	6.22	6.22	6.22	6.22	6.22
Calcium	0.84	0.84	0.84	0.84	0.84
Total phosphorus	0.58	0.58	0.58	0.58	0.58
Available phosphorus	0.44	0.43	0.42	0.41	0.40
Lysine	0.84	0.84	0.84	0.84	0.84
Methionine	0.38	0.38	0.38	0.38	0.38
Glucosinolate (μmol/g)	0	0.49	0.98	1.47	1.97

^1^ RSM, rapeseed meal; ME, metabolizable energy; RSM_4_, RSM_8_, RSM_12_, and RSM_16_ indicate that the levels of isonitrogenous replacement of soybean meal with RSM added to the diets were 4%, 8%, 12%, and 16%, respectively. Control group was fed a corn-soybean meal basal diet. ^2^ One kilogram of premix contained 1,200,000 IU retinol, 400,000 IU rachitasterol, 1800 IU D-a-tocopherol, 150 mg coagulation vitamin, 60 mg thiamine, 600 mg riboflavin, 200 mg pyridoxine, 1 mg cobalamin, 3 g nicotinic acid, 900 mg pantothenic acid, 50 mg folic acid, 4 mg biotin, 35 mg choline, 6 g Fe (ferrous sulfate), 1 g Cu (copper sulfate), 9.5 g Mn (manganese sulfate), 9 g Zn (zinc sulfate), 50 mg I (potassium iodide), and 30 mg Se (sodium selenite). ^3^ Calculated values.

**Table 3:** Effects of increasing dietary levels of rapeseed meal on the body weight (BW), average daily feed intake (ADFI), average daily gain (ADG), and feed/gain ratio (F/G) of geese.

Items ^2^	Groups ^1^					SEM	*p*-Value
	Control	RSM_4_	RSM_8_	RSM_12_	RSM_16_		
BW (g)							
35 d	1978	1988	1990	1988	1980	4.50	0.93
49 d	3008	3012	3031	3030	3032	12.16	0.96
70 d	3819	3849	3894	3870	3860	88.86	0.55
35 to 49 d							
ADFI (g/d/bird)	252.18	252.06	255.24	254.61	248.46	2.30	0.91
ADG (g/d/bird)	72.10	73.07	73.38	74.55	73.46	0.99	0.97
F/G (g/g)	3.52	3.46	3.48	3.43	3.38	0.03	0.79
49 to 70 d							
ADFI (g/d/bird)	256.82	252.74	261.98	255.66	258.50	2.60	0.32
ADG (g/d/bird)	38.6	39.85	41.11	40.00	39.80	0.57	0.47
F/G (g/g)	6.66	6.38	6.39	6.41	6.57	0.08	0.51
35 to 70 d							
ADFI (g/d/bird)	255.02	252.47	259.36	255.25	254.59	2.11	0.91
ADG (g/d/bird)	52.53	53.14	54.41	53.82	53.71	0.48	0.81
F/G (g/g)	4.86	4.76	4.77	4.74	4.74	0.03	0.84

^1^ RSM, rapeseed meal; RSM_4_, RSM_8_, RSM_12_, and RSM_16_ indicate that the levels of isonitrogenous replacement of soybean meal with RSM added to the diets were 4%, 8%, 12%, and 16%, respectively. Control group was fed a corn-soybean meal basal diet. ^2^ Each value represents the mean of six replicate pens.

**Table 4:** Effects of increasing dietary levels of rapeseed meal on carcass traits of geese at 70 d of age (%).

Items ^2^	Groups ^1^					SEM	*p*-Value
	Control	RSM_4_	RSM_8_	RSM_12_	RSM_16_		
Dressing percentage	89.99	88.08	88.83	87.65	88.37	0.52	0.70
Half-eviscerated carcass yield	76.37	74.94	75.84	74.73	75.24	0.50	0.81
Eviscerated carcass yield	81.04	80.09	80.31	79.39	79.90	0.45	0.86
Breast yield	2.50	2.55	2.54	2.56	2.59	0.15	1.00
Thigh yield	4.70	5.02	5.18	4.82	5.17	0.52	0.41
Abdominal fat yield	6.55	6.27	6.85	6.62	6.60	0.12	0.67

^1^ RSM, rapeseed meal; RSM_4_, RSM_8_, RSM_12_, and RSM_16_ indicate that the levels of isonitrogenous replacement of soybean meal with RSM added to the diets were 4%, 8%, 12%, and 16%, respectively. Control group was fed a corn-soybean meal basal diet. ^2^ Each value represents the mean of six replicate pens.

**Table 5:** Effects of increasing dietary levels of rapeseed meal on the relative weights of visceral organs of geese at 70 d of age.

Items ^2^	Groups ^1^					SEM	*p*-Value
	Control	RSM_4_	RSM_8_	RSM_12_	RSM_16_		
Heart	0.81	0.86	0.85	0.85	0.88	0.02	0.79
Liver	2.43	2.28	2.15	2.48	2.35	0.06	0.46
Spleen	0.13	0.12	0.10	0.12	0.13	0.01	0.84
Bursa of Fabricius	0.07 ^ab^	0.06 ^ab^	0.05 ^b^	0.06 ^ab^	0.08 ^a^	0.01	0.03
Gizzard	3.72	3.67	3.87	3.63	3.54	0.08	0.76
Proventriculus	0.36	0.35	0.34	0.35	0.35	0.01	0.96

^a,b^ In the same row, values with different letter superscripts mean significant difference (*p* < 0.05), while those with common or no letter superscript mean no significant difference (*p* > 0.05). ^1^ RSM, rapeseed meal; RSM_4_, RSM_8_, RSM_12_, and RSM_16_ indicate that the levels of isonitrogenous replacement of soybean meal with RSM added to the diets were 4%, 8%, 12%, and 16%, respectively. Control group was fed a corn-soybean meal basal diet. ^2^ Each value represents the mean of six replicate pens.

**Table 6:** Effects of increasing dietary levels of rapeseed meal on serum biochemical parameters of geese at 70 d of age ^1^.

Items ^3^	Groups ^2^					SEM	*p*-Value
	Control	RSM_4_	RSM_8_	RSM_12_	RSM_16_		
TP (g/L)	57.85	61.97	58.50	64.13	58.93	1.40	0.60
ALB (g/L)	24.70	26.00	24.26	24.48	24.70	0.37	0.63
GLU (mmol/L)	13.11	13.04	13.23	13.03	14.10	0.28	0.76
BUN (mmol/L)	0.93	0.99	0.89	0.87	0.76	0.04	0.47
TG (mmol/L)	0.65	0.79	0.69	0.67	0.63	0.03	0.66
CHO (mmol/L)	7.54	8.15	7.61	7.86	8.29	0.21	0.76
LDL (mmol/L)	2.57	2.82	2.71	2.91	2.74	0.09	0.87
HDL (mmol/L)	3.78	3.95	3.76	3.71	4.34	0.09	0.22
P (mmol/L)	3.67	3.97	3.58	4.02	3.40	0.09	0.22
Ca (mmol/L)	4.88	5.21	4.86	5.04	4.61	0.08	0.18
ALT (U/L)	25.78	29.90	26.00	24.05	22.53	1.15	0.33
AST (U/L)	118.5	105.4	116.9	99.19	99.62	7.40	0.88
AKP (U/L)	1512	1697	1704	1527	1447	56.34	0.52

^1^ Each value represents the mean of six replicate pens. ^2^ RSM, rapeseed meal; RSM_4_, RSM_8_, RSM_12_, and RSM_16_ indicate that the levels of isonitrogenous replacement of soybean meal with RSM added to the diets were 4%, 8%, 12%, and 16%, respectively. Control group was fed a corn-soybean meal basal diet. ^3^ TP, total protein; ALB, albumin; GLU, glucose; BUN, blood urea nitrogen; TG, triglyceride; CHO, cholesterol; LDL, low-density lipoprotein; HDL, high-density lipoprotein; P, phosphorus; Ca, calcium; ALT, alanine aminotransferase; AST, aspartate aminotransferase; AKP, alkaline phosphatase.

**Table 7:** Effects of increasing dietary levels of rapeseed meal on serum hormonal parameters of geese at 70 d of age ^1^.

Items ^3^	Groups ^2^					SEM	*p*-Value
	Control	RSM_4_	RSM_8_	RSM_12_	RSM_16_		
GH (ng/mL)	8.92 ^A^	9.34 ^A^	10.22 ^A^	13.66 ^B^	12.46 ^B^	0.42	<0.01
TSH (mU/L)	27.25	33.53	29.04	25.98	27.18	1.11	0.27
T3 (nmol/L)	5.19 ^ab^	5.00 ^b^	6.21 ^a^	5.89 ^ab^	5.88 ^ab^	0.15	0.05
T4 (nmol/L)	137.8	144.4	138.9	153.1	162.9	5.12	0.49

^A,B^ Means with different superscripts within the same row differ significantly (*p* < 0.01). ^a,b^ 0.05 ≤ *p* < 0.10 was considered to be a tendency towards significant. ^1^ Each value represents the mean of six replicate pens. ^2^ RSM, rapeseed meal; RSM_4_, RSM_8_, RSM_12_, and RSM_16_ indicate that the levels of isonitrogenous replacement of soybean meal with RSM added to the diets were 4%, 8%, 12%, and 16%, respectively. Control group was fed a corn-soybean meal basal diet. ^3^ GH, growth hormone; TSH, thyroid-stimulating hormone; T3, triiodothyronine; T4, tetraiodothyronine.

**Table 8:** Effects of increasing dietary levels of rapeseed meal on relative weight, villus height (μm) and crypt depth (μm) of geese at 70 d of age ^1^.

Items ^2^	Groups ^1^					SEM	*p*-Value
	Control	RSM_4_	RSM_8_	RSM_12_	RSM_16_		
Duodenum							
Relative weight	2.18	2.14	2.15	2.23	2.18	0.06	0.99
Villus height	1252	1215	1248	1162	1358	33.01	0.45
Crypt depth	298.3 ^a^	299.1 ^a^	297.4 ^a^	229.7 ^b^	233.5 ^b^	11.20	0.06
Jejunum							
Relative weight	4.74	4.19	4.35	4.17	4.67	0.11	0.40
Villus height	1363	1365	1524	1296	1483	43.72	0.47
Crypt depth	268.4	299.4	244.4	235.2	299.7	12.55	0.35
Ileum							
Relative weight	4.19	3.87	4.07	4.11	3.80	0.13	0.87
Villus height	1014	874.2	883.3	1009	783.8	39.20	0.29
Crypt depth	253.9	245.7	245.5	218.3	228.5	9.61	0.79

^a,b^ 0.05 ≤ *p* < 0.10 was considered to be a tendency towards significant. ^1^ Each value represents the mean of six replicate pens. ^2^ RSM, rapeseed meal; RSM_4_, RSM_8_, RSM_12_, and RSM_16_ indicate that the levels of isonitrogenous replacement of soybean meal with RSM added to the diets were 4%, 8%, 12%, and 16%, respectively. Control group was fed a corn-soybean meal basal diet.

## Data Availability

No other data reported.
